# Adolescent sexuality education, sexual debut, and associated factors in Nigerian public secondary schools

**DOI:** 10.1038/s41598-025-06360-8

**Published:** 2025-07-02

**Authors:** Tope Michael Ipinnimo, Olalekan Oladipupo Rosiji, Olumide Temitope Asake, Oluwafunmilayo Oluwadamilola Ibikunle, Motunrayo Temidayo Ipinnimo, Toluwalope Motunrayo Asake

**Affiliations:** 1 Department of Community Medicine, Federal Teaching Hospital, Ido-Ekiti, Nigeria; 2 Department of Community Medicine, Afe Babalola University, Ado-Ekiti, Nigeria; 3 Department of Obstetrics and Gynaecology, Federal Teaching Hospital, Ido-Ekiti, Nigeria; 4 Malaria Consortium, 33 Pope John Paul Street, Maitama, Abuja, Nigeria; 5 NHIA Department, Ekiti State University Teaching Hospital, Ado-Ekiti, Nigeria; 6 Department of Acute Medicine, Manchester Royal Infirmary, Manchester, UK

**Keywords:** Adolescent, Nigeria, Public secondary school, Sexual debut, Sexual intercourse, Sexuality education, Health care, Disease prevention, Health policy, Public health

## Abstract

Adolescents in Nigeria face significant sexual and reproductive health risks, yet the effectiveness of existing sexuality education programs remains uncertain. Addressing these gaps is critical to improving adolescent health outcomes. This study assessed sexuality education, sexual debut, and associated factors among public secondary school adolescents in Ekiti State, Nigeria. A cross-sectional study was conducted among 380 adolescents selected through multistage sampling. Data were collected using a validated semi-structured questionnaire covering sociodemographic variables, sexuality education (assessed with 13 questions exploring past sex-related discussions, sexually transmitted infection, contraceptive, and puberty), as well as engagement in sexual intercourse. Pearson chi-square and binary logistic regression were used to identify the predictors of engagement in sexual intercourse. Approximately 36.8% (*n* = 140) of respondents had a good level of sexuality education. Less than one-third, 27.1% (*n* = 103) had ever discussed sex-related matters with their parents or guardian. About 22.1% (*n* = 84) had engaged in sexual intercourse, with 41.7% (*n* = 35) having their sexual debut before the age of 15. Predictors of engagement in sexual intercourse included being ≥ 18 years old (AOR 12.881, 95% CI 3.615–45.892), male gender (AOR 2.573, 95% CI 1.353–4.892), senior secondary school enrollment (AOR 4.201, 95% CI 1.654–10.671), polygamous family background (AOR 2.508, 95% CI 1.007–6.316), having four or more siblings (AOR 3.778, 95% CI 2.043–6.986), and higher level of sexuality education (AOR 1.829, 95% CI 1.006–6.986). A huge proportion of adolescents who engage in sexual intercourse begin early; therefore, it is crucial to target interventions to address their needs at this stage. The limited parental involvement in sexuality education must be addressed to provide adolescents with safe, accurate, and socio-culturally sensitive comprehensive sexuality education. We suggest increasing advocacy to encourage parents and guardians to openly educate their children about sexuality.

## Introduction

Adolescence, defined by the World Health Organization (WHO) as the period between 10 and 19 years, is marked by significant physical, cognitive, and psychological changes, including the emergence of sexual and reproductive maturity^1,2^. These transitions can profoundly influence adolescents’ emotions, decision-making processes, and social interactions^1,2^. During this period, adolescents are particularly vulnerable to various sexual and reproductive health issues due to social influences, insufficient information, and limited access to healthcare services^3^. Consequently, they face increased risks of adverse outcomes such as early pregnancy, sexually transmitted infections (STIs), and school dropout^3,4^.

Given the potential risks and the high global population of this age group, the WHO emphasizes the importance of providing comprehensive, age-appropriate sexuality education to support their healthy growth and development^1^. Sexuality education equips young people with the knowledge and skills necessary to make informed decisions about sex and relationships throughout their lives^5^. This education further fosters the development of social and emotional competencies, which help young people build healthy relationships, critically analyze their surroundings, support marginalized individuals, and embrace self-love^5,6^.

Comprehensive sexuality education (CSE) aims to provide sound, up-to-date information needed to understand the reproductive system, gender, sexuality, life skills, STIs, and contraceptives to improve the health and well-being of people without bias to gender, age, and qualification^7^. It assists nations, communities, and families in giving information related to young people’s sexuality suitable for their developmental stage^7,8^. CSE utilizes informed decisions to prepare adolescents for intimate relationships and safe sex, leading to later onset of sexual activity, reduced risky behaviors, higher contraceptive use, and a safe, productive, fulfilling life against the odds of STIs, unintended pregnancies, gender-based violence, and gender inequality^8,9^.

In Nigeria, the integration of sexuality education into the school curriculum has faced inconsistencies due to sociocultural, religious, and policy-related challenges, despite the introduction of national sexuality education policies in 1999 and subsequent curriculum revisions. These barriers have hindered widespread acceptance and uniform delivery of sexuality education across the country, highlighting systemic gaps in the implementation and effectiveness of current educational programs^10,11^. Furthermore, Nigeria’s adolescent fertility rate remains one of the highest globally, with 104 births per 1000 women aged 15–19 years^12^.

Recent trends indicate a shift in adolescent sexual behaviour, with many young people engaging in sexual activity at increasingly younger ages due to earlier physical maturity^2,4,11^. This tendency is supported by global, regional, and local data^13–17^. The Nigeria demographic and health survey (NDHS) reveals that a significant proportion of adolescents initiate sexual activity before the age of 18, leading to an increase in unsafe sexual practices as they delay marriage^18,19^. Such sexual exposure increases the risks of multiple sexual partners, unprotected intercourse, contracting STIs such as Human Immunodeficiency Virus/Acquired Immunodeficiency Syndrome (HIV/AIDS), underage pregnancy, and unsafe abortion^20^.

Family dynamics and sociocultural factors play a crucial role in shaping adolescent sexual behaviour. Factors such as gender norms, family structure, peer influence, and exposure to sexual content through media significantly affect adolescents’ decisions regarding sexual activity. Studies have shown that adolescents from polygamous families, larger households, or those with limited parental involvement are more likely to engage in early sexual activity. Additionally, male adolescents often experience greater social freedom regarding sexual behaviour compared to their female counterparts^11,13,15–17^.

Although previous research in Nigeria has explored adolescent sexual behaviour and related risk factors, there remains a paucity of studies examining how the level of sexuality education interacts with these sociocultural determinants^4,6,18,19^. Understanding these relationships is essential for designing effective interventions that can address the root causes of risky sexual behaviour among adolescents. Also, existing literature has not adequately explored the combined effects of sociocultural factors and levels of sexuality education on adolescent sexual engagement in Nigeria. This study addresses this gap by evaluating how sexuality education, age at sexual debut, and various sociodemographic factors influence engagement in sexual intercourse among public secondary school students in Ekiti State.

This study aims to assess the level of sexuality education, the timing of sexual debut, and the factors associated with engagement in sexual intercourse among adolescents. The findings will provide evidence to inform educational policies and targeted interventions designed to promote safe sexual practices and contribute to achieving Sustainable Development Goal 3, which focuses on ensuring healthy lives and promoting well-being for all at all ages.

## Methods

### Study area and design

A quantitative analytical cross-sectional study design was adopted for this study as could capture multiple variables and the relationship between them at a snapshot and therefore, appropriate for answering the research question. The study was conducted between August and September 2021 in public secondary schools in a local government area (LGA) in Ekiti State, Southwest, Nigeria. The LGA has an average population of 239,600 people^21^. There are 15 public secondary schools in the area with a student population of about 7000. There is no health facility in the area providing adolescent-friendly healthcare services, however, guidance and counseling services are accessible to the students in their schools.

### Ethical approval

Ethical approval (protocol number: ERC/2021/07/23/613B) was obtained from the Human Ethics and Research Review Committee of the Federal Teaching Hospital, Ido-Ekiti. Permission was obtained from the school authorities before data collection. Respondents and their parents were given explanation on the nature, purpose, and benefits of the study and were made aware that they could withdraw at any time if they felt uncomfortable with the questionnaire’s content. Informed consent was obtained from parents of the adolescents and from those that are 18 years and above. Assents were sought from the other respondents before participating in the research. Anonymous self-administered questionnaire was used to collect data which was secured in a locked cabinet thereafter with access only to the principal investigator. All methods were performed in accordance with the relevant guidelines and regulations.

### Participants, sample size determination, and sampling technique

The study included all adolescent students (10–19 years) in public secondary schools in the area of study. Those who were absent from school on data collection day and those who joined the school less than 6 months before the study were excluded. The sample size for this study was estimated using the formula for qualitative outcome cross-sectional study^22^. A degree of accuracy, non-response rate and confidence interval of 5%, 10%, and 95%, respectively was assumed. A sample size of 380 was obtained after using the proportion of adolescents with sexual exposure in a previous study^23^ and adjusting for a population of less than 10,000 people.

A multistage sampling technique was adopted in selecting eligible respondents. In stage one, six out of 15 public secondary schools in the area were selected through simple random sampling by balloting without replacement. Also, stage 2 involved the selection through simple random sampling by balloting without replacement of one class arm from each class level (i.e. JSS1, JSS2, JSS3, SS1, SSS2, and SS3) in all six schools, making a total of 36 selected class arms. The balloting without replacement involves the selection of numbered folded similar papers corresponding to each category (school or class arm) from a basket without returning them to the pool. Lastly, a list of all adolescents in the selected class arm was generated as the sampling frame and systematic sampling was used to select eligible respondents. Since the populations of the selected schools and class arms were unequal, proportionate allocation was used to allot questionnaires at stages 1 and 2. The process of systematic sampling involves dividing the sampling frame by the allotted questionnaires to obtain the sampling interval that was used to select eligible adolescents. The first adolescent was selected using simple random sampling by balloting, after which subsequent adolescents were selected by adding sampling interval until the required respondents.

### Data collection procedure

The study instrument was a validated, semi-structured, self-administered questionnaire developed from the review of previous literature^2,3,11,20^. The questionnaire obtained data on sociodemographic variables, level of sexuality education (13 questions exploring past sex-related discussions, STIs, contraceptives, and puberty), engagement in sexual intercourse, and age at sexual debut. The questionnaire was reviewed for clarity, relevance, and comprehensiveness by a clinical epidemiologist, consultant public health physicians, consultant obstetrician and gynecologist, as well as sexual and reproductive health experts, to ensure face and content validity. Pre-testing was done among 40 adolescents in a public secondary school in another LGA in Ekiti State about 30 km away and necessary corrections were made after the pre-testing. The items in the questionnaire were tested for internal consistency and Cronbach’s Alpha score was 0.78.

### Measures

Thirteen questions assessed the level of sexuality education and were answered with a Yes or No option. Out of the 13 questions, six were positively worded and seven were negatively worded. For the positively worded questions, the correct answer was “Yes”, while for the negatively worded questions, “No” was the correct response. Correct and wrong answers were assigned 1 and 0 points, respectively making 13 the highest obtainable score and 0 the lowest obtainable score. Respondents were categorized into good (≥ 50% of the highest obtainable score) and poor (< 50% of the highest obtainable score) levels of sexuality education based on their scores.

Engagement in sexual intercourse (sexual debut) was assessed with a question inquiring if the adolescent had ever had sexual intercourse (insertion and thrusting of the penis inside the vagina).

### Variables and data analysis

The independent variables in this study included sociodemographic characteristics and sexuality education, while the dependent variable included engagement in sexual intercourse among adolescents. The independent variables in this study were selected based on empirical evidence from previous studies showing their potential association with engagement in sexual intercourse among the adolescent age group. Several studies have identified sociodemographic characteristics such as age, gender, and class, among others, as predictors of adolescent sexual behavior^11,15–17^. These variables were chosen not only because of their established relevance in the literature but also for their alignment with this study’s objective as well as contextual appropriateness for the target population.

Data analysis was carried out using IBM SPSS software for Windows, Version 26.0. (IBM Corp., Armonk, NY, USA). Frequency and percentages (categorical variables), as well as mean and standard deviation (*SD*) (numerical variables), were used to present descriptive statistics. For inferential statistics, Pearson chi-square and binary logistic regression analysis were used to test for significance at the bivariate and multivariate levels of analysis, respectively, comparing the association between engagement in sexual intercourse and independent variables. A *p* value < 0.05 was considered significant.

## Results

A total of 380 respondents completed the survey. Among this sample, half (50.0%, *n* = 190) were < 15 years and the male-to-female ratio was 0.8:1. About two-thirds (57.6%, *n* = 219) of the respondents were in senior secondary class. The majority of the respondents are from a monogamous family (90.0%, *n* = 342) and are living with their parents (93.7%, *n* = 356). More than one-third (35.3%, *n* = 134) of respondents were firstborns and a little above half (50.8%, *n* = 193) had at least 4 siblings. The majority (86.1%, *n* = 327) had their parents living together. Details of sociodemographic variables is in Table 1.

**Table 1:** Sociodemographic variable of respondents (*N* = 380).

Variable	Frequency (*n*)	Percent (%) [95% confidence interval]
Age group (in years)		
< 15	190	50.0 [45.0–55.0]
15–17	165	43.4 [38.4–48.4]
≥ 18	25	6.6 [4.1–9.1]
Gender		
Male	169	44.5 [39.5–49.5]
Female	211	55.5 [50.5–60.5]
Class		
JSS 1–3	161	42.4 [37.4–47.3]
SSS 1–3	219	57.6 [52.7–62.6]
Type of family		
Monogamous	342	90.0 [87.0–93.0]
Polygamous	38	10.0 [7.0–13.0]
Who you live with		
Parents	356	93.7 [91.2–96.1]
Guardian/relative	24	6.3 [3.9–8.8]
Parents living together		
Yes	327	86.1 [82.6–89.5]
No	53	13.9 [10.5–17.4]
Position in the family		
1st	134	35.3 [30.5–40.1]
2nd	86	22.6 [18.4–26.8]
3rd	82	21.6 [17.4–25.7]
4th	53	13.9 [10.5–17.4]
5th	25	6.6 [4.1–9.1]
Number of siblings		
1–3	187	49.2 [44.2–54.2]
≥ 4	193	50.8 [45.8–55.8]

More than half (57.1%, *n* = 217) of respondents had discussed sex-related matters with an adult or counselor in the past while less than one-third (27.1%, *n* = 103) had ever discussed sex-related matters with their parents or guardian. About a third (34.7%, *n* = 132) believed a woman cannot get pregnant the first time she had sexual intercourse, 38.7% (*n* = 147) believed a woman is unlikely to get pregnant if she had sexual intercourse halfway between her periods, 45.0% (*n* = 171) had the notion that condoms are not effective ways of protecting against sexually transmitted diseases, while 69.2% (*n* = 263) believed that sexually transmitted infections are contracted through unprotected sex. Details of responses to exposure to sexuality education among the respondents are in Table 2. Table 3 shows that about one-third (36.8%, *n* = 140) of the respondents had a good level of sexuality education with a mean (± *SD*) sexuality education score of 40.7 (± 15.6).

**Table 2:** Sexuality education among the respondents (*N* = 380).

Variable	Yes *n* (%)	No *n* (%)
An adult/counselor has discussed sex-related matters with me before^a^	217 (57.1)	163 (42.9)
You have discussed sex-related matters with your parents or guardian^a^	103 (27.1)	277 (72.9)
A woman cannot get pregnant the first time she has sexual intercourse^b^	132 (34.7)	248 (65.3)
A woman is unlikely to get pregnant if she had sexual intercourse halfway between her periods^b^	147 (38.7)	233 (61.3)
Condoms are not effective ways of protecting against sexually transmitted diseases^b^	171 (45.0)	209 (55.0)
Sexually transmitted infections are contacted through unprotected sex^a^	263 (69.2)	117 (30.8)
Contraceptive pills can prevent sexually transmitted infection^b^	115 (30.3)	265 (69.7)
Condoms are not protective against pregnancy^b^	101 (26.6)	279 (73.4)
Menstruation is not a sign of sexual maturity^b^	89 (23.4)	291 (76.6)
Menstruation starts at the age of 9-13^a^	183 (48.2)	197 (51.8)
Deepening of voice is not a sign of sexual maturity^b^	120 (31.6)	260 (68.4)
Do you see beards as a sign of sexual maturity^a^	188 (49.5)	192 (50.5)
Growth of axillary hair signifies sexual maturity^a^	198 (52.1)	182 (47.9)

^a^Positively worded question.

^b^Negatively worded question.

**Table 3:** Level of sexuality education among respondents (*N* = 380).

Variable	Frequency (*n*)	Percent (%) [95% confidence interval]
Level of sexuality education		
Good	140	36.8 [32.0–41.9]
Poor	240	63.2 [58.3–68.0]
Mean sexuality education score ± SD (%)	40.7 [95% CI 39.1–42.3] ± 15.6	

In Table 4, 84 (22.1%) respondents had been engaged in sexual intercourse. Out of these, 84, about two-fifths (41.7%, *n* = 35) had a sexual debut < 15 years of age while almost half (48.8%, *n* = 41) of them had their sexual debut between 15 and 17 years. The mean age (± *SD*) at sexual debut was 15.2 (± 1.5) years.

**Table 4:** Engagement in sexual intercourse and age at sexual debut (*N* = 380).

Variable	Frequency (*n*)	Percent (%) [95% confidence interval]
Sexual intercourse		
Yes	84	22.1 [17.9–26.3]
No	296	77.9 [73.7–82.1]
Age at sexual debut (years) *n* = 84		
< 15	35	41.7 [31.1–52.2]
15–17	41	48.8 [38.1–59.5]
≥ 18	8	9.5 [3.3–15.8]
Mean age ± SD of sexual debut	15.2 [95% CI 14.9–15.5] ± 1.5	

Table 5 shows statistically significant associations between engagement in sexual intercourse and age (*p* < 0.001), gender (*p* < 0.001), class (*p* < 0.001), type of family (*p* = 0.002), number of siblings (*p* = 0.007) as well as sexuality education (*p* = 0.010). There was a higher proportion of older students, males, those in the senior classes, those from polygamous families, students who had more siblings, and those with a good level of sexuality education who had engaged in sexual intercourse than their counterparts.

**Table 5:** Factors associated with engagement in sexual intercourse (*N* = 380).

Variables	Sexual intercourse (*n*)		Chi-square test	*p* value
	Yes *n* = 84 (%)	No *n* = 296 (%)		
Age group (years)				
< 15	14 (7.4)	176 (92.6)	73.564	**< 0.001**
15–17	51 (30.9)	114 (69.1)		
≥ 18	19 (76.0)	6 (24.0)		
Gender				
Male	52(30.8)	117 (69.2)	13.268	**< 0.001**
Female	32 (15.2)	179 (84.8)		
Class				
JSS 1–3	9 (5.6)	152 (94.4)	44.252	**< 0.001**
SSS 1–3	75 (34.2)	144 (65.8)		
Type of family				
Monogamous	68 (19.9)	274 (80.1)	9.808	**0.002**
Polygamous	16 (42.1)	274(57.3)		
Who you live with				
Parents	76 (21.3)	280 (78.7)	1.876	0.171
Guardian/relative	8 (33.3)	16 (66.7)		
Parents living together				
Yes	73 (22.3)	254 (77.7)	0.065	0.798
No	11 (20.8)	42 (79.2)		
Position in the family				
1st	28 (20.9)	106 (79.1)	5.323	0.365
2nd	8 (9.3)	78 (90.7)		
3rd	13 (15.9)	69 (84.1)		
4th	26 (49.1)	27 (50.9)		
5th	9 (36.0)	16 (64.0)		
Number of siblings				
1–3	30 (16.0)	157 (84.0)	7.859	**0.007**
≥ 4	54 (28.0)	139 (72.0)		
Level of sexuality education				
Good	41 (29.3)	99 (70.7)	6.637	**0.010**
Poor	43 (17.9)	197 (82.1)		

Bold indicates significant values (*p* < 0.05).

Table 6 shows the predictors of engagement in sexual intercourse among the respondents. Respondents who were ≥ 18 years old were about 13 times more likely to engage in sexual intercourse than those aged < 15 years (AOR 12.881, 95% CI 3.615–45.892). Males were about two and a half times more likely to be sexually exposed than females (AOR 2.573, 95% CI 1.353–4.892). Additionally, students in senior secondary school were about 4 times more likely to engage in sexual intercourse than students in junior secondary school (AOR 4.201, 95% CI 1.654–10.671). Students from polygamous families were 2.5 times more likely to be sexually exposed than those from monogamous families (AOR 2.508, 95% CI 1.007–6.316). Likewise, respondents who had at least four siblings were about 4 times more likely to engage in sexual intercourse than those with lesser siblings (AOR 3.778, 95% CI 2.043–6.986). Lastly, respondents who had a higher sexuality education were about 2 times more likely to engage in sexual intercourse than those with a lower level of sexuality education (AOR 1.829, 95% CI 1.006–6.986).

**Table 6:** Binary logistic regression for the predictors of engagement in sexual intercourse (*N* = 380).

Variables	Adjusted odd ratio	95% confidence interval		*p* value
		Lower	Upper	
Age group (years)				
< 15 (reference)	1.000			
15–17	2.175	0.913	5.180	0.079
≥ 18	12.881	3.615	45.892	**< 0.001**
Gender				
Male	2.573	1.353	4.892	**< 0.001**
Female (reference)	1.000			
Class				
JSS 1–3 (reference)	1.000			
SSS 1–3	4.201	1.654	10.671	**0.004**
Type of family				
Monogamous (reference)	1.000			
Polygamous	2.508	1.007	6.316	**0.049**
Number of siblings				
1–3 (reference)	1.000			
≥ 4	3.778	2.043	6.986	**< 0.001**
Level of sexuality education				
Good	1.829	1.006	6.986	**0.048**
Poor (reference)	1.000			

Bold indicates significant values (*p* < 0.05).

## Discussion

The study conducted an empirical analysis of adolescent sexuality education, sexual debut, and the factors associated with engagement in sexual intercourse among Nigerian public secondary school adolescents. A little above half of the respondents had discussed sex-related matters with an adult or a counsellor. This is lesser than findings from Rivers State, Nigeria, where the majority of participants had received sexuality education from an adult and school authority^24^. Another work in PortHacourt, Rivers State, Nigeria showed that four-fifths of adolescents had discussed or read about sexual matters; however, relatively half of them had discussed sexual issues with relations or parents^4^. These trends show that sexuality education provided by adults or counselors is lower in this current study compared to the aforementioned^4,24^; however, there is an obvious similarity when taking into context the source of the education.

Furthermore, this study revealed that the vast majority of adolescents had a poor level of sexuality education. This finding is similar to those reported in Ondo State, Nigeria^25^. Also, the finding aligns with a United Nations Educational, Scientific and Cultural Organization (UNESCO) report indicating that only 37% of young people in sub-Saharan Africa possess comprehensive knowledge about HIV transmission and prevention^26^. This finding underscores the need for more effective and comprehensive sexuality education for adolescents to address and eradicate the negative consequences of poorly informed sexual relationships.

In this study, parental contribution to sexuality education is minimal. Similarly, a study in Tanzania found that about six out of ten respondents did not discuss sexual-related matters with their parents or guardians when they were adolescents, meanwhile, they would have loved to^27^. This unmet need may be attributed to parents’ reluctance to discuss such sensitive topics with their children^28^. Parents are among the first and most influential sources of sexual information for adolescents. From an early age, adolescents form attitudes about gender roles, relationships, and sexual norms through interactions with their caregivers. The nature and extent of parental communication about sexuality can significantly impact adolescents’ sexual knowledge, behaviors, and attitudes^29^. Parents who engage in open, honest discussions about sex provide their children with a foundation for making informed decisions about relationships and sex. Conversely, parents who avoid or only engage in restrictive conversations about sexuality may inadvertently contribute to confusion, misinformation, and early sexual activity^30^. A positive parental approach to sexuality education should involve discussions that are age-appropriate, balanced, and supportive, fostering an environment where adolescents feel comfortable seeking guidance when needed.

This study found that about one-fifths of the respondents have had sexual intercourse, corroborating the findings of another study^11^, with about four out of every ten of them initiating before the age of 15 and another five out of every ten initiating between the ages of 15 and 17. This finding is slightly different from previous research, which reported that around 60% of respondents had sexual intercourse before the age of 15^11^. This suggests that public health measures aimed at reducing the negative impact of inadequate sexuality education would be most effective if implemented during early adolescence^31^.

Age 18 years and above, being in senior secondary class, male gender, coming from a polygamous family type, and having more siblings were identified as predictors of engagement in sexual intercourse during adolescence. Past studies have also demonstrated a similar association between these variables—age, class, gender, and engagement in sexual intercourse^11,32–39^. These adolescent variables could be valuable in identifying at-risk populations, helping to target public health interventions more effectively.

The age at which adolescents engage in sexual intercourse is influenced by a combination of biological, psychological, and social factors. Older adolescents are more likely to engage in sexual activity, as physical and emotional maturation increase sexual desire and readiness^33^. Age is also interrelated with school class, as adolescents in senior secondary classes are more likely to be older and mature, providing a possible explanation for why they are more likely to engage in sexual intercourse. Puberty accelerates sexual activity, as adolescents may experience increased curiosity and opportunities for sexual behavior^34^. Younger adolescents may be less equipped to navigate the emotional and physical consequences of sexual intercourse. Early initiation of sexual activity is associated with higher risks of poor sexual health outcomes, including STIs and teenage pregnancy^35^. Conversely, late initiation may reflect a combination of social and personal factors, including parental monitoring and stronger academic engagement^36^.

Gender plays a significant role in the timing and likelihood of sexual activity during adolescence. Adolescent girls and boys often have different motivations, risks, and outcomes related to sexual activity^37,38^. Boys tend to report earlier initiation of sexual activity, possibly due to societal expectations of male sexual aggression and early engagement in sexual behaviors. In contrast, girls are more likely to experience pressure regarding sexual behavior, either through peer influence or societal expectations of femininity and modesty^37,38^. Adolescent girls may face unique challenges such as higher exposure to sexual coercion, sexual violence, and unintended pregnancies, making them more vulnerable to the consequences of early sexual intercourse^39^. Also, gendered expectations in certain cultures, most especially those that place a high value on female virginity, may discourage earlier sexual initiation among females, thereby affecting the timing and context in which sexual experiences occur^40^.

Another predictor of sexual intercourse in this study was sexuality education. It was revealed that a good level of sexuality education increases the odds of engaging in sexual intercourse among adolescents. According to the WHO, comprehensive sexuality education focused on guiding adolescents towards healthy intimate relationships, safe sexual practices, and a productive, fulfilling life through informed decisions^8,9^. Although a higher sexuality education predicts a higher likelihood of sexual intercourse in this study, we did not explore the nature of sexual intercourse among the adolescents; it is possible they acted on informed decisions and made the right choices of having safe sex. This is one of the limitations of this current study and an area for future research.

Additionally, this study is also limited by social desirability bias as questions were related to the sexual behavior of adolescents, as well as recall bias as inquiries were made about their past experiences. Recall bias was reduced by carefully phrasing and avoiding leading questions. Social desirability bias was minimized by anonymity and confidentiality in the survey process. The questionnaire was self-administered with no identifiable questions, such as names and addresses. We acknowledged that sociocultural and economic factors, such as poverty, peer pressure, and deeply rooted cultural norms, which may influence sexual behaviour, especially among adolescents in Africa, were not examined in this study. Future research should explore the broader socio-economic and cultural factors that may influence adolescents’ sexual behaviour, employing mixed-method approaches.

## Conclusions

The lack of adequate sexuality education for adolescents is largely due to the limited involvement of parents in the study area. There is a clear unmet need that must be addressed to provide adolescents and young adults with safe, accurate, and comprehensive sexuality education. Although a significant number of adolescents have discussed sexual matters with an adult or counsellor, the vast majority still receive inadequate sexuality education. It is evident that a huge proportion of adolescents who engage in sexual intercourse begin during early adolescence; therefore, it is crucial to target interventions to address their needs at this stage.

We recommend increasing advocacy to encourage parents and guardians to openly educate their children about sexuality. Additionally, the content and methods used by counsellors in delivering this crucial education should be reviewed to ensure adolescents are well informed and educated about sexuality, thereby reducing the potential negative outcomes of inadequate sexuality education.

## Data Availability

All data generated or analysed during this study are included in this published article.

## Abbreviations

AIDS: Acquired Immunodeficiency syndrome
CSE: Comprehensive sexuality education
HIV: Human immunodeficiency virus
LGA: Local government area
NDHS: Nigeria demographic and health survey
SD: Standard deviation
STI: Sexually transmitted infections
UNESCO: United Nations Educational, Scientific and Cultural Organization
WHO: World Health Organization
